# Study of the Influencing Factors of Cyberbullying Among Chinese College Students Incorporated With Digital Citizenship: From the Perspective of Individual Students

**DOI:** 10.3389/fpsyg.2021.621418

**Published:** 2021-03-04

**Authors:** Jinping Zhong, Yunxiang Zheng, Xingyun Huang, Dengxian Mo, Jiaxin Gong, Mingyi Li, Jingxiu Huang

**Affiliations:** School of Educational Information Technology, South China Normal University, Guangzhou, China

**Keywords:** cyberbullying, college student, influencing factors, digital citizenship, individual students

## Abstract

Understanding the influencing factors of cyberbullying is key to effectively curbing cyberbullying. Among the various factors, this study focused on the personal level of individual students and categorized the influencing factors of cyberbullying among college students into five sublevels, i.e., background, Internet use and social network habits, personality, emotion, and literacy related to digital citizenship. Then a questionnaire survey was applied to 947 Chinese college students. The results show that cyberbullying among Chinese college students are generally at a low level. There are many factors influence cyberbullying. Specifically, at the personal background level, gender has a significant impact on cyberbullying and being cyberbullied. In terms of personal Internet use and social network habits, students’ average daily online time has no significant correlation with cyberbullying and being cyberbullied; however, the proportion of online non-learning time has a significantly positive correlation with cyberbullying, and the proportion of online learning/work time has a significant impact on being cyberbullied. At the personality level, the Big Five personality traits have varying degrees of correlation with and influence on cyberbullying and being cyberbullied. At the personal emotions level, students’ life satisfaction has a significantly negative correlation with cyberbullying and being cyberbullied while it only has a significant impact on being cyberbullied; the personal stress and empathetic concern aspects of empathy have a significantly positive correlation with cyberbullying and being cyberbullied among female students. At the literacy related to digital citizenship level, students’ understanding of and compliance with Internet etiquette have significantly negative impacts on cyberbullying; the ability to communicate and collaborate online and Internet addiction have significantly positive impacts on cyberbullying and being cyberbullied; the understanding of and compliance with relevant digital laws and regulations have significantly negative correlations with cyberbullying and being cyberbullied. Overall, college students’ digital citizenship level has a significantly negative correlation with cyberbullying but no significant correlation with being cyberbullied. Finally, analysis and suggestions were provided according to these statistical results and the effects of these factors on cyberbullying and being cyberbullied among college students, so as to help solve this problem and provide a new perspective for research in this field.

## Introduction

Currently, the Internet has penetrated into all aspects of people’s lives. While providing various conveniences, the Internet has also caused a series of social problems such as spam, Internet addiction, and Internet crime. In recent years, cyberbullying, as a representative of abnormal Internet behaviors, has been prominent in many countries (e.g., the United States, Japan, and Australia), in which countermeasures and preventive measures against cyberbullying have been formulated. Instagram, a well-known social platform, began developing automated cyberbullying filtering tools in 2019. In his book, Ivester (2011) maintains that social media is evolving into an alternative mechanism of communication and contact among people and is continuously in fashion among students, greatly increasing the likelihood of cyberbullying on college campuses (Washington, 2015). This is especially true for Chinese college students. Statistical results show that Internet users aged 10–19 and 20–29 accounted for 14.8 and 19.9% of the whole population in China (China Internet Network Information Center, 2020), and 87.8% of college students love to use social communication applications (iiMedia Research, 2018). Partly because Chinese college students have much free time and are curious about the outside world, which, coupled with the absence of parental supervision, has led to college students being the major Internet users among the adolescent population. However, negative information is becoming more common in digital society. Being inexperienced and immature emotionally and intellectually, without having established the “Three Views”^1^, college students are more inclined to be inadvertently involved in cyberbullying (as a perpetrator or a victim) and exert adverse influences on others and society as a whole.

Under this circumstance, it is necessary to know the current situation of cyberbullying among Chinese college students and reveal potential influencing factors to help cub it effectively. However, the literature survey of the China National Knowledge Infrastructure (CNKI) indicated that as of July 2020, there has been only 13 publications on “cyberbullying” and “influencing factors,” all published after 2015, accounting for 3.8% of all 337 articles with the subject “cyberbullying.” The lack of studies on the influencing factors of cyberbullying makes relevant prevention strategies and containment mechanisms ineffective and impertinent. Additionally, in terms of research objects, most of the previous studies in China have focused on cyberbullying among youth, with only 32 articles on college students and none on influencing factors. In fact, college life is the most critical time before an individual enters society and thus a critical period for the formation and establishment of personality, morals, and the “Three Views.” Being deeply involved in the Internet and digital society, college students should be guided to keep away from cyberbullying. Therefore, understanding the influencing factors of cyberbullying among them and developing targeted prevention strategies are very important for effectively addressing the problem. In this regard, based on discovering the current situation of college student cyberbullying in China, this paper examined its influencing factors from the perspective of individual students to provide suggestions for the intervention and prevention of cyberbullying.

## Literature Review and Hypotheses

### Literature Review

Literature review showed that the existing studies mainly focused on individual students, families, schools, society, and the environment. Specifically, in terms of individual students, Li (2007), Kowalski et al. (2012b), Topcu and Erdur-Baker (2012) and many other investigators revealed that cyberbullying is gender related. Hsu and Wang (2010) found that personality traits are predictive of cyberbullying, and Gibb and Devereux (2014) and Goodboy and Martin (2015) showed that the dark personality theory can describe the common characteristics of cyberbullies: self-righteous, ruthless, and aggressive. From the psychological perspective, Sun and Deng (2016) found that both perpetrators and victims of cyberbullying have more negative emotions; Liu and Xu (2019) found that the psychological factors related to cyberbullying include empathy, narcissism, self-esteem, depression, and anxiety; Gini and Pozzoli (2009) and Renati et al. (2012) found that cyberbullying is associated with an individual’s empathy; cyberbullying perpetrators often lack empathy and have emotional difficulties (Weaver and Lewis, 2012; Barlińska et al., 2013). Zhao and Wang (2019) demonstrated that college students’ perception of well-being is closely correlated with their Internet usage, and Li (2007), You (2013), Hayton (2017), and Nurlita et al. (2018) showed that the frequencies of Internet use and social media use have an important impact on cyberbullying.

In terms of family factors, Ybarra and Mitchell (2004) found that cyberbullying is closely related to the relationship between family members; Wang et al. (2012), Bayraktar et al. (2015), and Elsaesser et al. (2017) confirmed the connection between cyberbullying behavior and a lack of parental support; and Pillay (2012) and Park et al. (2014) found that cyberbullying is associated with individuals’ family socioeconomic status to some extent. In addition, some studies revealed that parental supervision is also a factor affecting cyberbullying (Ybarra and Mitchell, 2004; Chen and Astor, 2012; Kowalski et al., 2012a; Low and Espelage, 2013).

Regarding school factors, Bevilacqua et al. (2017) showed that the degree of cyberbullying varies with school type and quality, and organizational/management factors within a school affect students’ behavior; Guarini et al. (2012) found that students’ negative relationship with teachers and low recognition of the school are risk factors for cyberbullying; and Calvete et al. (2010) and Souza et al. (2018) found that cyberbullying is related to school atmosphere and environment. Moreover, school culture (Monks et al., 2016), safety (Bottino et al., 2015) and regulatory measures (Song, 2015), sense of belonging (Baldry et al., 2015; Chen et al., 2016), and education and training on mental health and cybersecurity (Gao, 2018; Liang, 2019) are also important factors affecting cyberbullying.

With respect to social and environmental factors, Huang and Chou (2010) argued that cyberbullying behaviors, in various countries, are highly dependent on the environment and are affected by the education system, school environment, cultural norms, and interpersonal relationships. Markward et al. (2001) found that various factors, such as herd mentality, traditional bullying influence, and cultural background differences, affect cyberbullying behavior. In addition, workplace stress (Vranjes et al., 2017) and peer factors (Liu and Xu, 2019) are also related to the risk of cyberbullying among youth, which is also affected by the characteristics of the Internet (Kiesler et al., 1985; Holland, 2012).

In recent years, digital citizenship education has gradually attracted widespread attention from scholars around the world. With the aim of cultivating qualified digital citizens in the information age, digital citizenship education requires digital citizens to acquire global awareness, legal awareness as well as digital citizenship awareness so that technology is used in a safe, responsible, and ethical way (Yang et al., 2016). However, the rise and spread of cyberbullying are inextricably linked to each digital citizen: current Internet users are mostly digital natives who have acquired the ability to use information technology but still lack the corresponding technical ethics and responsibilities. In other words, the occurrence of many cyberbullying incidents is the outcome of weak cyber legal and moral awareness among these digital natives. That’s exactly the core of digital citizenship education (Ivester, 2011; Zheng et al., 2020). Therefore, while providing a new perspective for the study of cyberbullying, digital citizenship education is an important means to control cyberbullying (Lin, 2017; Zheng et al., 2020). In this regard, digital citizenship, in conjunction with the relevant digital citizenship education content were investigated in this study to conduct an in-depth examination on the influencing factors of cyberbullying at the personal level.

The above literature review and analysis categorizes the influencing factors of cyberbullying into four levels: (1) Personal level, including gender, age, personality traits, well-being, empathy, length or frequency of Internet uses, social behavior type, and digital citizenship; (2) Family level, including relationship between family members, parental support, family socioeconomic status, and parental supervision; (3) School level, including school type and teaching quality, school management, teacher-student relationship, school climate and environment, school culture, school safety and supervision, and education and training on mental health and Internet security; (4) Social and environmental level, including national education system, cultural norms, community influence (herd mentality), cultural differences, interpersonal (peer) relationship, work pressure, and Internet characteristics.

Among the above-described influencing factors, those at students’ personal level have a direct impact on students’ cyberbullying behavior, and are the basis for investigating and analyzing the influencing factors of cyberbullying at other levels. So it sounds reasonable to start from the perspective of individual students. Nevertheless, previous studies have focused on students’ personal variables (e.g., gender, age or grade, and personality traits) and Internet usage (e.g., hours online and frequency per day), without considering students’ literacy related to digital citizenship. Therefore, in this study, personal influencing factors of cyberbullying among college students were categorized into five sublevels, i.e., (1) Background (including gender, age, and time to start using the Internet), (2) Internet use and social network habits (including average daily time online, the proportion of online learning/non-learning time, the number of online social communities joined, and social behavior type), (3) Personality [including five personality traits, i.e., openness, neuroticism, extroversion, agreeableness, and conscientiousness (Howard et al., 1996)], (4) Emotion (including subjective well-being and empathy), and (5) Literacy related to digital citizenship [including digital identity and dignity, digital citizenship awareness and accountability, the understanding of and compliance with Internet etiquette, digital communication and collaboration capabilities, degree of Internet addiction, and the understanding of and compliance with relevant laws and regulations (Ribble, 2015; Zheng et al., 2020)].

### Hypotheses

In order to explore the impact of personal factors on cyberbullying, this study inspected these variables one by one, as illustrated in the following hypotheses:

Hypothesis 1: The degree of cyberbullying among Chinese college students is affected by students’ personal background. Specifically, college students of different genders and with different ages to start using the Internet have significantly different scores regarding the degree of cyberbullying. This hypothesis corresponds to exploring the influence of individual background (sublevel 1) on cyberbullying.
Hypothesis 2: The degree of cyberbullying among Chinese college students is affected by students’ use of the Internet and social network habits. Specifically, cyberbullying among college students has a significantly positive correlation with students’ length of time online and the proportion of online non-learning time, and students who show different social network habits differ significantly regarding cyberbullying. This hypothesis corresponds to exploring the influence of individual Internet use and social network habits (sublevel 2) on cyberbullying.
Hypothesis 3: The degree of cyberbullying among Chinese college students is affected by students’ personality traits. Specifically, the degree of cyberbullying has a significantly positive correlation with neuroticism and openness but a significantly negative correlation with extroversion, agreeableness, and conscientiousness. This hypothesis corresponds to exploring the influence of individual personality (sublevel 3) on cyberbullying.
Hypothesis 4: The degree of cyberbullying among Chinese college students is affected by students’ emotions. Specifically, the degree of cyberbullying has a significantly negative correlation with their life satisfaction and empathy. This hypothesis corresponds to exploring the influence of individual emotion (sublevel 4) on cyberbullying.
Hypothesis 5: The degree of cyberbullying among Chinese college students is affected by students’ level of digital citizenship and has a significantly positive correlation with their degree of Internet addiction and a significantly negative correlation with their digital identity and dignity, digital citizenship awareness and accountability, understanding of and compliance with Internet etiquette, digital communication and collaboration skills, and understanding of and compliance with relevant laws and regulations. This hypothesis corresponds to exploring the influence of individual literacy related to digital citizenship (sublevel 5) on cyberbullying.

## Research Design and Implementation

### Research Subjects and Process

In this study, through random sampling, college students and graduate students of different cities in China took part in this online survey anonymously. Specifically, a text message and a questionnaire link were first sent to the students of South China Normal University randomly via social communication software (e.g., WeChat groups, QQ groups), then they were asked to forward the message to their classmates or ex-classmates (e.g., their high school classmates but now learning in different universities). Gradually the survey was spread out in a non-linear way. Each student was asked to provide responses to the survey within a specified time. Since ethical review and approval is not required for the study on human participants in accordance with the local legislation and institutional requirements of China, an instruction about the purpose of this survey and how the data will be used later was provided at the beginning of the questionnaire, so that the participants had a total understanding of the survey. Eventually a total of 1,188 online questionnaires were collected, of which 947 were valid, for an effective rate of 79.7%.

### Questionnaire Design

The questionnaire consisted of five parts:

(1) Questions regarding students’ personal background, Internet use and social network habits, including students’ gender, age, time to start using the Internet, average daily time online, proportion of online learning/non-learning time, number of online social communities joined, and types of social behavior, in a total of seven items. In China, students mainly use popular social networking platforms such as Sina Microblog, Tencent Microblog, QQ Groups, WeChat Groups, Tianya social community, Zhihu social community, and the like. Of course, some of them may use Facebook, Instagram, Twitter or similar platforms. They will all be considered by default when it comes to statistical analysis of one’s online social networking experience. This instruction was also provided in the questionnaire to make students clearly understand.
(2) A personality questionnaire, i.e., The Big Five Personality Test, compiled by Howard et al. (1996) and used to measure the personality inclination of college students, in a total of 25 items. This questionnaire has been widely used in many studies, with high reliability and validity [0.736 < Cronbach’s α < 0.904 and KMO = 0.806 (Hee, 2014)].
(3) Emotion questionnaires to analyze subjective well-being and empathy, measured, respectively, with the Life Satisfaction Scale developed by Diener et al. (1985) and the Interpersonal Reactivity Index scale compiled by Davis (1980). Both scales have been tested and have good reliability and validity [Cronbach’s α = 0.86 and KMO = 0.84 for the Life Satisfaction Scale (Silva et al., 2015) and Cronbach’s α = 0.75 and KMO = 0.833 for the Interpersonal Reactivity Index Scale (Zhang et al., 2010)]. There are totally 27 items in this part.
(4) A digital citizenship questionnaire that measures, using 35 questions answered with a five-point Likert scale, digital identity and dignity, digital citizenship awareness and accountability, the understanding of and compliance with Internet etiquette, digital communication and collaboration capabilities, degree of Internet addiction, and the understanding of and compliance with relevant laws and regulations. Among them, the Internet Addiction Scale was derived from the simplified version of Young’s Internet Addiction Test with high reliability and validity [Cronbach’s α = 0.848 and KMO = 0.924 (Pawlikowski et al., 2013)], the scales for the rest variables were modified from or developed based on, respectively, the self-esteem scale for the assessment of adolescents’ self-worth and self-acceptance by Rosenberg (1965), the digital citizenship scale (Al-Zahrani, 2015), the monograph on digital citizenship education by Ribble (2015) and the content decomposition of digital citizenship by Zheng et al. (2020). The whole questionnaire in this part was tested in this study and found to have good reliability and validity (Cronbach’s α = 0.789 and KMO = 0.671).
(5) A cyberbullying questionnaire derived from Topcu and Erdur-Baker’s (2010) Cyberbullying Scale that measures the degree to which college students act as perpetrators or victims of cyberbullying. The questionnaire uses 14 items for 14 cyberbullying behaviors, with another 14 for being cyberbullied behaviors. So totally there are 28 items, with high reliability and validity [Cronbach’s α = 0.818 and KMO = 0.873 (Murwani, 2019)]. In order to get a better understanding of how personal factors have influence on cyberbullying among college students, the questionnaire limits cyberbullying experience (commit or suffer) to be within the recent one or 2 years. In other words, students will be asked if they have had these experiences (14 cyberbullying behaviors and 14 being cyberbullied behaviors) recently.

## Results

### Descriptive Statistics

**FIGURE 1:**
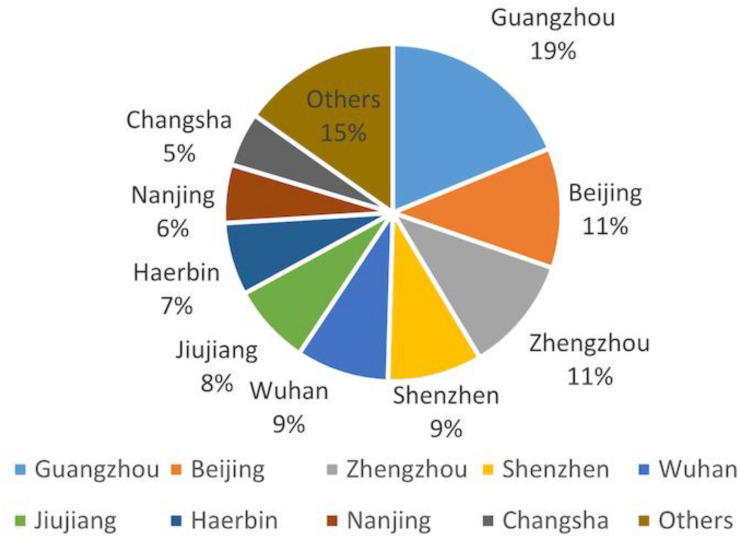
Geographical distribution of the respondents.

Figure 1 shows the geographical distribution of the respondents. It’s clear that the participants were mostly from big and modern cities of China, such as Guangzhou, Beijing, Zhengzhou, and Shenzhen, where Internet access is easier and faster, and social network application is more popular as well.

The respondents’ demographic information, Internet use and social network habits are shown in Table 1. They were young people with an average age of 20.71 (SD = 2.234). Two-thirds of them were female, indicating that in China girls showed more willingness to help others academically than boys. Over one-half of the respondents (53.9%) started their online experience prior to middle school; on average, 45.2% of the students spent 3–6 h online daily, and one-third of the students spent over 6 h online daily. College students spent an average of 66.63% of time online on social networks and doing other activities unrelated to learning. When using social networks, 54.1% of the students joined at least three online communities while 65.3% did not participate in any online discussions.

**TABLE 1:** Statistics for college students’ background information, Internet use and social network habits.

Category	Level	Frequency	Percentage (%)
Gender	Male	305	32.2
	Female	642	67.8
Age	19 years or younger	254	26.8
	20 years	264	27.9
	21 years	170	18.0
	22 years	105	11.1
	23 years or older	154	16.3
Time to start using the Internet	Pre-school	34	3.6
	Elementary school	476	50.3
	Middle school	324	34.2
	College	102	10.8
	Other	11	1.2
Average daily time online	Less than 1 h	12	1.3
	1–3 h	179	18.9
	3–6 h	428	45.2
	6–10 h	258	27.2
	Over 10 h	70	7.4
Number of online communities joined	0	117	12.4
	1–3	317	33.5
	3–6	231	24.4
	7–10	95	10.0
	Over 10	187	19.7
Types of online social behavior	Self-expressive	46	4.9
	Socially active	160	16.9
	Participates in discussions	123	13.0
	Does not participate in discussions	618	65.3

### Current Situation of Cyberbullying Among College Students

According to Topcu and Erdur-Baker’s (2010) Cyberbullying Scale, the total score ranges from 14 to 56 points. The higher the score is, the higher the level of cyberbullying or being cyberbullied. As shown in Table 2, overall, the average cyberbullying score for the 947 college students was 17.14, indicating a low cyberbullying level; the average score for being a victim of cyberbullying was 19.93, which is low but higher than that for cyberbullying. Among the 14 cyberbullying behaviors, “Making fun of comments in online forums” appeared most frequently in both situations (*M* = 2.20 and SD = 1.319 for cyberbullying, and *M* = 1.88 and SD = 1.201 for being cyberbullied), while “Excluding others by blocking or moving their comments” (*M* = 1.87 and SD = 1.077) and “Stealing email access (usernames and passwords) and blocking true owner’s access” (*M* = 1.84 and SD = 0.999) ranked second in frequently appeared forms of cyberbullying and being cyberbullied, respectively.

**TABLE 2:** Statistics for cyberbullying among college students.

	Range	Min.	Max.	*M*	SD
Cyberbullying	23	14	37	17.14	3.431
Being cyberbullied	36	14	50	19.93	6.239

According to Brack and Caltabiano (2014), when committing (suffering) any of the 14 behaviors two or more times, an individual can be deemed as a cyberbullying perpetrator (victim). Those with a dual identity of cyberbullying perpetrator and victim must meet the standards for a cyberbullying perpetrator and victim simultaneously while those who are deemed as non-participants either never committed or experienced any cyberbullying or experienced one incident, at most, of cyberbullying or being cyberbullied. According to these criteria, the proportion of college students who are cyberbullying victims (58.6%) is a bit higher than that of students who are cyberbullying perpetrators (51.2%), and more than 40% of them have a dual identity as both a victim and perpetrator (41.6%); approximately one-third of the students have never experienced cyberbullying (31.8%). Though results show high percentages of cyberbullying and being cyberbullied (over 50%), the most frequent form of both cyberbullying and being cyberbullied is making fun of comments on forums (it’s very common in this era), and the average scores are 17.14 and 19.93 (out of 56), respectively, with SD less than 2. Therefore, it is believed that cyberbullying is generally at a relatively low level among Chinese college students, so is being cyberbullied.

### Influencing Factors of Cyberbullying Among College Students

#### Effect of Personal Background on Cyberbullying

##### Gender

Gender differences in cyberbullying were examined through the two independent samples non-parametric test. As shown in Table 3, the progressive significance values are lower than 0.05, indicating that gender differences in cyberbullying is significant. The scores for male students are significantly higher than those for female students, indicating that male students are more likely to cyberbully others or be cyberbullied by others than are female students.

**TABLE 3:** Significance tests for gender differences in cyberbullying.

	Cyberbullying		Being cyberbullied	
Gender	Male	Female	Male	Female
Number of cases	305	642	305	642
Average score	18.36	16.56	22.25	18.83
Mann–Whitney *U* statistics	70550.000		68003.500	
Sig. (progressive significance)	0.000		0.000	

##### Time to start using the Internet

The relationship between the time to start using the Internet and cyberbullying was examined through the two independent samples non-parametric test. As shown in Table 4, the progressive significance values are lower than 0.05, indicating that students with different ages to start using the Internet differ significantly regarding cyberbullying.

**TABLE 4:** Significance tests for time to start using the Internet in cyberbullying.

	Cyberbullying					Being cyberbullied				
Time to start using the Internet	1^a^	2	3	4	5	1	2	3	4	5
Number of cases	34	476	324	102	11	34	476	324	102	11
Average score	18.62	17.58	16.46	16.64	18.00	23.76	20.46	18.79	19.74	20.73
χ2	30.699					24.036				
Sig.	0.000					0.000				

*^*a*^Kruskal–Wallis test. 1 = Pre-school, 2 = Elementary school, 3 = Middle school, 4 = College, 5 = Other.*

#### Effect of Internet Use and Social Network Habits on Cyberbullying

##### Internet use

The correlation between the degree of cyberbullying and daily average time online or daily average non-learning time online was analyzed using the Spearman correlation method. As shown in Table 5, daily average time online is not significantly correlated to cyberbullying while daily non-learning time online is significantly positively correlated with the degree of cyberbullying but is not significantly correlated with the degree of being cyberbullied.

**TABLE 5:** Correlation between Internet use and cyberbullying.

		Cyberbullying	Being cyberbullied
Daily average time online	Spearman correlation coefficient	0.062	0.038
	Sig. (two-tailed)	0.058	0.248
	Number of cases	947	947
Proportion of daily non-learning time online	Spearman correlation coefficient	0.073*	−0.025
	Sig. (two-tailed)	0.025	0.440
	Number of cases	947	947

*p < 0.05; the same below.

##### Social network behavior

The effect of social behavior type on the degrees of cyberbullying and being cyberbullied was analyzed through variance analysis. As shown in Table 6, the significance values are all lower than 0.05, indicating that different social behaviors have significant effects on cyberbullying among college students.

**TABLE 6:** Variance analysis results for the effect of social behavior type on cyberbullying.

Types of social behavior	Cyberbullying			Being cyberbullied		
	*M*	SD	Sig.	*M*	SD	Sig.
Self-expressive	17.91	3.681	0.000	22.04	7.800	0.002
Socially active	17.67	3.469		20.65	6.736	
Participates in discussions	18.02	3.540		20.93	6.542	
Does not participate in discussions	16.77	3.326		19.39	5.841	

#### Effect of Personality Traits on Cyberbullying

The relationship between the personality traits of college students and cyberbullying behavior was examined through the Big Five Personality Test and Spearman correlation analysis. As shown in Table 7, the degree of cyberbullying is significantly positively correlated with openness and significantly negatively correlated with neuroticism, agreeableness and conscientiousness. The degree of being cyberbullied is significantly positively correlated with openness, and significantly negatively correlated with neuroticism and conscientiousness.

**TABLE 7:** Correlation between Big Five personality traits and cyberbullying.

			Cyberbullying	Being cyberbullied
Spearman’s	Neuroticism	Correlation coefficient	−0.157**	−0.129**
rho		Sig. (two-tailed)	0.000	0.000
		*N*	947	947
	Extroversion	Correlation coefficient	−0.018	−0.011
		Sig. (two-tailed)	0.588	0.730
		*N*	947	947
	Openness	Correlation coefficient	0.139**	0.080*
		Sig. (two-tailed)	0.000	0.014
		*N*	947	947
	Agreeable ness	Correlation coefficient	−0.094**	−0.035
		Sig. (two-tailed)	0.004	0.278
		*N*	947	947
	Conscientiousness	Correlation coefficient	−0.175**	−0.109**
		Sig. (two-tailed)	0.000	0.001
		*N*	947	947

*p < 0.05; **p < 0.01.

#### Effect of Emotions on Cyberbullying

##### Life satisfaction

The results of the Spearman correlation between life satisfaction and cyberbullying/being cyberbullied are shown in Table 8, indicating that students’ life satisfaction is negatively correlated with the degree of cyberbullying as well as with the degree of being cyberbullied.

**TABLE 8:** Correlation between life satisfaction and cyberbullying.

			Cyberbullying	Being cyberbullied
Spearman’s rho	Life satisfaction	Correlation coefficient	−0.106**	−0.090**
		Sig. (two-tailed)	0.001	0.005
		*N*	947	947

**p < 0.01.

##### Empathy

Given the gender differences in empathy, the samples were grouped based on two genders, and Spearman correlation between empathy and cyberbullying was conducted for the two groups, respectively. As shown in Table 9, the correlation between each of the empathy variables and cyberbullying (or being cyberbullied) is non-significant in the male student group while the personal distress and empathetic concern variables of empathy are significantly positively correlated with both cyberbullying and being cyberbullied in the female student group.

**TABLE 9:** Correlation between empathy and cyberbullying.

Spearman			Cyberbullying	Being cyberbullied
Male	Empathy-personal distress	Correlation coefficient	0.082	0.029
		Sig. (two-tailed)	0.152	0.478
		*N*	305	305
	Empathy-perspective taking	Correlation coefficient	−0.076	0.011
		Sig. (two-tailed)	0.185	0.781
		*N*	305	305
	Empathy-fantasy	Correlation coefficient	0.002	0.072
		Sig. (two-tailed)	0.970	0.084
		*N*	305	305
	Empathy-Empathetic concern	Correlation coefficient	−0.019	0.038
		Sig. (two-tailed)	0.745	0.361
		*N*	305	305
Female	Empathy-personal distress	Correlation coefficient	0.113**	0.100**
		Sig. (two-tailed)	0.004	0.001
		*N*	642	642
	Empathy-perspective taking	Correlation coefficient	−0.057	−0.022
		Sig. (two-tailed)	0.150	0.452
		*N*	642	642
	Empathy-fantasy	Correlation coefficient	0.042	0.043
		Sig. (two-tailed)	0.293	0.138
		*N*	642	642
	Empathy-Empathetic concern	Correlation coefficient	0.083*	0.066*
		Sig. (two-tailed)	0.035	0.024
		*N*	642	642

*p < 0.05; **p < 0.01.

#### Effect of Digital Citizenship on Cyberbullying

The effect of digital citizenship on cyberbullying among college students was examined through the Spearman correlation of cyberbullying with students’ digital identity and dignity, digital citizenship awareness and accountability, understanding of and compliance with Internet etiquette, digital communication and collaboration capabilities, and understanding of and compliance with relevant laws and regulations. As shown in Table 10, the average scores for all variables related to college students’ digital citizenship (except Internet addiction) are higher than 10; that for students’ understanding of and compliance with relevant laws and regulations is the highest, and that for students’ digital communication and collaboration capabilities is the lowest. The correlation analysis results showed that the degrees of cyberbullying and being cyberbullied are significantly positively correlated with students’ digital communication and collaboration capabilities, and are significantly negatively correlated with students’ understanding of and compliance with relevant laws and regulations; whereas only the degree of cyberbullying is significantly negatively correlated with students’ understanding of and compliance with Internet etiquette. In general, students’ level of digital citizenship is significantly negatively correlated with the degree of cyberbullying but is not significantly correlated with the degree of being cyberbullied.

**TABLE 10:** Statistics for students’ digital citizenship and correlations between students’ digital citizenship and cyberbullying.

Variable	Range	Min.	Max.	*M*	SD	Correlation with cyberbullying		Correlation with being cyberbullied	
						Correlation coefficient	Sig. (Two-tailed)	Correlation coefficient	Sig. (Two-tailed)
Digital communication and collaboration capabilities	10	7	17	11.56	1.760	0.191**	0.000	0.174**	0.000
Digital identity and dignity	16	4	20	17.10	2.376	0.026	0.420	−0.027	0.398
Digital citizenship awareness and accountability	12	8	20	15.91	1.623	−0.027	0.398	−0.007	0.827
Understanding of and compliance with Internet etiquette	16	4	20	16.99	2.320	−0.156**	0.000	−0.042	0.200
Understanding of and compliance with relevant laws and regulations	19	6	25	20.32	2.397	−0.127**	0.000	−0.076*	0.020
Digital citizenship level	45.89	18	63.89	51.10	5.754	−0.138**	0.000	−0.052	0.112

*p < 0.05; **p < 0.01.

In order to reveal the relationship between Internet addiction and cyberbullying, the Internet addiction status of Chinese college students was first analyzed, then followed by the correlation between Internet addiction and cyberbullying/being cyberbullied through Pearson correlation analysis. For the Internet Addiction Scale, the higher the score is, the higher the degree of Internet addiction; a score above 40 indicates an Internet addiction. As shown in Tables 11, 12, 19.3% of the students are addicted to the Internet, and the students’ Internet addiction is significantly positively correlated with the degree of cyberbullying or being cyberbullied, indicating that the higher the degree of a student’s Internet addiction, the more likely that student is to commit cyberbullying or be cyberbullied.

**TABLE 11:** Internet addiction among college students.

	Total score		Frequency		Percentage
Internet addiction	<40 (no Internet addiction)		764		80.7
	>40 (Internet addiction)	40–60 (mild)	182	183	19.3
		60–80 (moderate)	1		
		80–100 (severe)	0		
	*M*		32.97		
	SD		7.518		

**TABLE 12:** Correlation between Internet addiction and cyberbullying among college students.

		Cyberbullying	Being cyberbullied
Internet addiction	Pearson correlation	0.217**	1**
	Sig. (two-tailed)	0.000	0.000
	Number of cases	947	947

**p < 0.01.

#### Multivariate Regression Analysis of Influencing Factors of Cyberbullying

To further examine the joint effects of these personal factors on cyberbullying among college students, multivariate regression analyses were conducted using the above variables as independent variables and the degrees of cyberbullying and being cyberbullied as dependent variables; the samples were grouped based on social behavior type, with the socially active group as the reference group and students who do not participate in discussions (accounting for 65.3% of the total sample) as an example in the analysis.

As shown in Table 13, after excluding several non-significant variables based on the *F*-test, nine predictors remained in the regression equation for cyberbullying factors, each having a tolerance greater than 0.4 and a VIF value below 5, indicating that these nine predictors retained in the regression equation do not have a multicollinearity problem. The significance of the *F* value (sig.) is lower than 0.001, indicating that these predictors have a significant linear relationship with the degree of cyberbullying. Specifically, at the personal background level, gender has a significant impact on the degree of cyberbullying. At the Internet use and social network habits level, social behavior type and the number of online communities joined have significant impacts on the degree of cyberbullying. At the personality trait level, only conscientiousness has a significantly positive impact on the degree of cyberbullying, while other traits were eliminated in the stepwise linear regression, indicating that other aspects of the Big Five personality traits have no significant linear relationships with the degree of cyberbullying. At the digital citizenship level, Internet addiction, digital communication and collaboration capabilities, and digital citizenship awareness and accountability have significantly positive impacts on the degree of cyberbullying, while students’ understanding of and compliance with Internet etiquette has a significantly negative impact on the degree of cyberbullying.

**TABLE 13:** Results of the multivariate regression analysis of factors influencing the degree of cyberbullying in students who do not participate in online discussions.

Model	Unstandardized coefficient		Standardized coefficient	*t*	Significance	Collinearity statistics	
	*B*	SE	Beta			Tolerance	VIF
(Constant)	11.151	1.393		8.003	0.000		
Gender (reference group: male)	−1.610	0.223	−0.219	−7.223	0.000	0.938	1.066
Time to start using the Internet (reference group: before middle school)	0.909	0.944	0.028	0.963	0.336	0.995	1.005
Social behavior type (reference group: socially active)	−0.515	0.222	−0.072	−2.325	0.020	0.914	1.095
Number of online communities joined	0.237	0.080	0.091	2.979	0.003	0.938	1.066
Conscientiousness	0.133	0.036	0.114	3.743	0.000	0.925	1.081
Internet addiction level	0.088	0.014	0.193	6.279	0.000	0.917	1.091
Digital communication and collaboration capabilities	0.259	0.060	0.133	4.302	0.000	0.909	1.101
Understanding of and compliance with Internet etiquette	−0.213	0.049	−0.144	−4.333	0.000	0.783	1.278
Digital citizenship awareness and accountability	0.177	0.070	0.084	2.536	0.011	0.799	1.252

*R = 0.434; *R*^2^ = 0.189; adjusted *R*^2^ = 0.181; *F* = 24.220; Sig. < 0.001.*

In the stepwise multivariate regression equation for factors influencing the degree of being cyberbullied, ten predictors remained in the equation, each having a tolerance greater than 0.4 and a VIF value below 5, showing no multicollinearity problem between the variables. The significance of the *F* value (sig.) is lower than 0.001, indicating that these predictors have a significant linear relationship with the degree of being cyberbullied. As shown in Table 14, at the personal background level, gender has a significant impact on the degree of being cyberbullied. At the Internet use and social network habits level, the number of online communities joined and online learning/work time has significant impacts on the degree of being cyberbullied. At the emotion level, life satisfaction has a significantly negative impact on the degree of being cyberbullied. At the personality level, conscientiousness has a significantly positive impact on the degree of being cyberbullied. At the digital citizenship level, the degree of Internet addiction, digital communication and collaboration capabilities, and digital identity and dignity have significantly positive impacts on the degree of being cyberbullied.

**TABLE 14:** Results of the multivariate regression analysis of factors influencing the degree of being cyberbullied in students who do not participate in online discussions.

Model	Unstandardized coefficient		Standardized coefficient	*t*	Significance	Collinearity statistics	
	*B*	SE	Beta			Tolerance	VIF
(Constant)	6.197	2.183		2.839	0.005		
Gender (reference group: male)	−3.317	0.407	−0.249	−8.152	0.000	0.961	1.041
Time to start using the Internet (reference group: before middle school)	0.986	1.747	0.017	0.565	0.572	0.992	1.008
Social behavior type (reference group: socially active)	0.585	0.411	0.045	1.422	0.155	0.905	1.105
Number of online communities joined	0.319	0.147	0.067	2.171	0.030	0.942	1.061
Online learning/work time	0.032	0.011	0.087	2.814	0.005	0.934	1.070
Life satisfaction	−0.092	0.034	−0.086	−2.670	0.008	0.868	1.152
Conscientiousness	0.149	0.067	0.070	2.230	0.026	0.898	1.114
Internet addiction level	0.148	0.027	0.178	5.549	0.000	0.871	1.148
Digital communication and collaboration capabilities	0.507	0.111	0.143	4.548	0.000	0.905	1.105
Digital identity and dignity	0.181	0.081	0.069	2.238	0.025	0.941	1.063

*R = 0.405; *R*^2^ = 0.164; adjusted *R*^2^ = 0.155; *F* = 18.324; Sig. < 0.001.*

## Discussion

This study randomly selected 947 college students in China as survey subjects to investigate the current situation of cyberbullying and conducted an in-depth analysis on the impact of students’ personal background, Internet use and social network habits, personality traits, emotions and literacy related to digital citizenship on the degrees of cyberbullying and being cyberbullied. Further analysis and discussions are presented as follows.

### Effect of Student’s Personal Background on Cyberbullying Among College Students

Regarding gender, the male students’ total scores for cyberbullying and being cyberbullied were significantly higher than those for the female students, indicating that males are more likely to cyberbully others or be cyberbullied by others than are females, which is consistent with the results of some previous studies (Calvete et al., 2010; Huang and Chou, 2010; Ozden and Icellioglu, 2014; Safaria, 2016; Beyazit et al., 2017) but contrary to those of others (Smith et al., 2008; Ortega et al., 2009; Sourander et al., 2010; Giménez-Gualdo et al., 2015), likely because in different countries, regions or schools, the understanding and identification of cyberbullying differ, and there are many measurement scales in this field, in which certain behaviors deemed as cyberbullying are controversial. On the other hand, the Internet use awareness and online behavior of different survey subjects vary and are closely related to their education and experience from childhood onward. In addition, the methods for cyberbullying commonly used by male and female students also differ (Slonje and Smith, 2008; Wong et al., 2014). Therefore, there are three different conclusions regarding the effect of gender on cyberbullying: more males commit cyberbullying, more females commit cyberbullying, and both genders commit cyberbullying equally (Hinduja and Patchin, 2008; Guarini et al., 2012; Pillay, 2012; Gibb and Devereux, 2014). Therefore, this remains an open question. In regard to the participants in this study, male students had stronger personalities and were more volatile than female students and thus more inclined to have conflicts with others, leading to cyberbullying (Zhu et al., 2016).

In addition, time to start using the Internet is significantly correlated with students’ cyberbullying or being cyberbullied, but the two showed no regression relationship, which is likely related to the students’ Internet awareness, skills and experience. Early exposure to the Internet allows students to have stronger Internet use awareness, more Internet skills and richer Internet experience, making these students more adept to cyberspace and prone to bully newbies intentionally or unintentionally. On the other hand, the participation of college students have been growing in various online forums and communities, which, in the early stage, were relatively open and laden with all kinds of information for which effective supervision and reporting mechanisms lacked; therefore, the longer a student has had access to the Internet (i.e., the earlier the time to start using the Internet), the more cyberbullying the student would have suffered.

These results confirm Hypothesis 1 listed in section “Hypotheses,” suggesting that in cyberbullying intervention and governance processes, it is necessary to pay close attention to the social behavior of male students, especially those with an early age to start using the Internet.

### Effect of Students’ Internet Use and Social Network Habits on Cyberbullying

Regarding average daily time online, though daily time online is not correlated with cyberbullying, daily non-learning time online is significantly positively correlated (but no regression relationship) with the degree of cyberbullying, and the proportion of learning/work time online has a significant regression relationship with the degree of being cyberbullied. In other words, the longer the daily non-learning time a student spends online, the more likely he/she is to become a perpetrator of cyberbullying; the longer the daily learning/work time a student spends online, the more likely he/she is to become a cyberbullying victim. In previous studies, time online was not divided into learning and non-learning hours, but cyberbullying usually occurs in non-learning situations, such as social interactions, games, and entertainment; therefore, the conclusions of this study can be considered consistent with those of previous studies (Hinduja and Patchin, 2008; Sticca et al., 2013; Zhu et al., 2016). This result indicates that students with different purposes and uses for the Internet have different effects on others. Lingering on social network and leisure sites makes these students more susceptible to disinformation or misinformation, prompting them to use offensive and threatening language, send tasteless pictures that violate others’ privacy, or place blame on teammates when playing online games, thereby cyberbullying others.

In terms of social behavior, different types of online behavior are significantly correlated with cyberbullying or being cyberbullied. Regarding average cyberbullying scores, students who are self-expressive and participate in discussions are more inclined to cyberbully others. Students with these two behaviors belong to active social network types and are prone to voice their views and follow suit when participating in debates; when questioned or refuted or when questioning or debating others, these students are liable to have conflict with others and even engage in cyber-stalking and violate the privacy of others, thereby cyberbullying others. Regarding average scores for being cyberbullied, students who are self-expressive had significantly higher scores than those of students with other behaviors, indicating that those who like to voice their opinions and ideas online are more likely to be cyberbullied, especially when their opinions or views are not accepted by others.

These results mostly confirm Hypothesis 2, suggesting that in the cyberbullying intervention and governance processes, it is necessary to strictly control the non-learning/work hours of college students and treat those with different social behaviors differently, so that targeted measures can be taken to prevent cyberbullying.

### Effect of College Students’ Personality on Cyberbullying

First, the personality trait “openness” is significantly positively correlated with cyberbullying and being cyberbullied, i.e., college students with a high level of openness are more likely to cyberbully others or be cyberbullied, which is consistent (Hsu and Wang, 2010; You, 2013; Peluchette et al., 2015) or partially consistent (Celik et al., 2012) with the results reported in other studies, indicating that these students are curious about the outside world, fond of trying new things and thus more prone to be involved in Internet events or comment on others’ opinions, leading to online conflicts. Moreover, students with a high degree of openness have more Internet interactions on a wider range of topics and thus are more prone to be exposed to misinformation or disinformation while fully exposing their own information on the Internet, making them more susceptible to cyberbullying.

Second, neuroticism and conscientiousness are significantly negatively correlated with students’ cyberbullying and being cyberbullied, i.e., college students with strong neuroticism and those who are conscientious are less likely to cyberbully others or be cyberbullied, which is consistent (Festl and Quandt, 2013; You, 2013) or partially consistent (Celik et al., 2012) with the results of other studies, indicating that college students who can more effectively balance emotions, such as anxiety and hostility, maintain emotional stability and are more organized, with a greater sense of responsibility and self-control, are less likely to exhibit cyberbullying behaviors and be cyberbullied.

Third, agreeableness is significantly negatively correlated with cyberbullying, i.e., college students with a high level of agreeableness are less likely to cyberbully others, which is consistent with the result of a previous study (Celik et al., 2012). Students with a high level of agreeableness give priority to others, get along with others well and interact with others more harmoniously and thus are popular among others; they are often friendly and considerate and rarely bully others online. However, agreeableness is not significantly correlated with being cyberbullied, which is inconsistent with the findings of other studies (Celik et al., 2012; You, 2013; Semerci, 2017), likely because students with a high level of agreeableness are always ready to help others and friendly to others; therefore, they are less likely to become a target of bullying by others.

These results partly confirm Hypothesis 3, suggesting that in cyberbullying intervention and governance processes, it is necessary to first determine a student’s personality traits and propose specific measures for college students with different personalities, and if conditions permit, big data and data mining techniques can be employed to determine their personality traits and predict cyberbullying behavior more accurately.

### Effect of Students’ Emotions on Cyberbullying

Students’ life satisfaction is significantly negatively correlated with cyberbullying and being cyberbullied and has a significant impact on being cyberbullied, indicating that the higher the level of students’ life satisfaction, the less likely the students will bully others or be bullied, which is consistent with the results of a previous study (Zhu et al., 2016) but different from those of another study (Pillay, 2012); this inconsistency is likely due to the differences between college students in China and other countries when perceiving happiness and the aspects different assessment scales focusing on.

In terms of empathy, personal stress, and empathic concern are significantly positively correlated with cyberbullying and being cyberbullied among female students; however, this correlation is absent among male students, indicating that gender plays a mediating role in the effect of empathy on cyberbullying, which is consistent with the results of some early studies (Topcu and Erdur-Baker, 2012; Baldry et al., 2015; Del Rey et al., 2016) but contrary to those of other studies (Renati et al., 2012; Brewer and Kerslake, 2015; Peterson and Densley, 2017). These inconsistent results are likely due to the differences in the active areas of male and female brains regarding displaying empathy (Schulte-Rüther et al., 2008); the emotional awareness of females is stronger, making them more inclined to sympathize and emphasize with others’ stress and perceive and understand others by taking the position of others, ultimately resulting in “being involved too deeply to be able to disengage” and thus being more susceptible to being cyberbullied. They may also turn empathy into vengeance and condemn those who they consider perpetrators through inappropriate ways, such as breeching privacy, verbal abuse and insults, turning a self-righteous act into cyberbullying.

These results mostly confirm Hypothesis 4, suggesting that in cyberbullying intervention and governance processes, it is necessary to pay attention to students’ life satisfaction as well as the emotional stability of female students and integrate Internet supervision mechanism to dynamically display students’ emotional data so that cyberbullying behaviors can be accurately monitored and prevented.

### Effect of College Students’ Literacy Related to Digital Citizenship on Cyberbullying

In the first place, students’ understanding of and compliance with Internet etiquette has a significantly negative impact on cyberbullying, indicating that college students’ understanding and recognition of digital ethics, such as Internet etiquette and technical etiquette, actively practicing positive ethics and codes of conduct in the digital space, and regulating their behaviors in digital society through etiquette in real society can allow the vast majority of people to enjoy the convenience and joy brought by digital technology and effectively reduce the probability of cyberbullying. Therefore, it is advisable to fully acknowledge the advantages of school, family and community education, improve college students’ awareness of Internet etiquette, expand the Internet etiquette knowledge base, and cultivate relevant operational skills and norms in all life aspects through supplementation with various lifelong education models, coupled with related online and offline promotion to effectively improve college students’ understanding of and compliance with Internet etiquette, so as to effectively prevent cyberbullying.

In the second place, college students’ digital communication and collaboration capabilities have a significantly positive impact on cyberbullying and being cyberbullied. Cyberbullying mainly manifests as verbal abuse with insulting and offensive language, or privacy disclosures. The results showed that college students who are more able to skillfully select appropriate means of communication and collaboration with others online are more adept at mastering a variety of communication means and skills; once their emotions are out of control, they are prone to voice some inappropriate opinions or disclose the privacy of others, thus resulting in cyberbullying. On the other hand, college students with digital communication and collaboration capabilities are more likely to join more online communities, have richer online social networks or collaboration experience and spend longer amounts of time online, increasing their likelihood of being cyberbullied. Therefore, it is necessary to supervise and control the time and space of communication and collaboration; in particular, schools and families should pay special attention to those students with strong digital communication and collaboration capabilities, and when necessary, administrative and technical means should be used to strictly manage their social networks and collaborations to prevent cyberbullying incidents.

In the third place, college students’ degree of Internet addiction has a significantly positive impact on cyberbullying and being cyberbullied, indicating that students who are more addicted to the Internet are more dependent on the Internet, resulting in higher probabilities of cyberbullying others and being cyberbullied, which is consistent with the results of earlier studies (Floros et al., 2013; Chang et al., 2015; Hou, 2017). College students are not fully mature mentally, are profoundly affected by emotions and have not yet formed the “Three Views”; when lingering online for too long, they are vulnerable to mental, emotional, and moral erosion through misinformation and disinformation on the Internet and thus develop negative behaviors, intentionally or unintentionally cyberbullying others or being cyberbullied by others. Therefore, it is necessary to pay attention to their digital health and wellness; in schools and families, when necessary, administrative and technical means should be utilized to strictly monitor and control their online time, establish an early warning mechanism for excessive Internet use and take various anti-addiction measures to prevent Internet addiction, encouraging them to find a balance between online and offline life.

In the fourth place, college students’ understanding of and compliance with relevant digital laws and regulations are significantly negatively correlated with cyberbullying and being cyberbullied, indicating that the understanding of and compliance with laws and policies on technology use, especially rules related to Internet ethics, digital rights and responsibilities in the form of legal regulations (e.g., copyright protection for intellectual property), are particularly important for college students’ online behavior. These laws and regulations restrict and regulate the online behaviors, allowing them to clearly know which behaviors are illegal in digital society so that they can strictly abide by them, which helps to significantly reduce the probability of cyberbullying and being cyberbullied. Therefore, it is necessary to strengthen college students’ knowledge and understanding of relevant digital laws and regulations through education at schools, in families and in the community, guiding them to use information technology legally and regulating their words and actions online to avoid cyberbullying and being cyberbullied.

In general, the level of digital citizenship is significantly negatively correlated with the degree of cyberbullying but is not significantly correlated with the degree of being cyberbullied, indicating that improving college students’ digital citizenship level can help significantly reduce their likelihood of cyberbullying others, which mostly confirms Hypothesis 5. Digital citizenship is about the values, necessary qualities, key abilities, and behavior habits for using technology safely, legally, and ethically (Hao, 2014; Zheng et al., 2020). Improving college students’ literacy related to digital citizenship will definitely lead to their mastery of knowing how to use technology legally and ethically in daily learning and life, so that the probability of cyberbullying and being cyberbullied among college students can be reduced, and the harm to individuals’ body and mind as well as to society can be avoided, which will ultimately purify cyberspace to a certain extent and prompt the formation of a healthy cyber civilization. Education departments and schools should emphasize and strengthen college students’ digital citizenship education to enhance their digital citizenship in all aspects, thereby ensuring better survival and development in the digital world.

## Conclusion

While bringing convenience to people’s interactions, the Internet also causes an obscuration of values and a deficiency in subjectivity (Hao, 2014). It has been well established that cyberbullying has become one of the increasingly serious social problems in the Internet era. Preventing cyberbullying not only relies on means that emphasize “blocking” approaches, such as traditional Internet monitoring, regulations, and legislation, but also requires the adoption of “dredging” approaches to guide youth to correct online behaviors and improve their digital citizenship level, which is also one of the main objectives of digital citizenship education (Lin, 2017; Zheng et al., 2020). Incorporated with digital citizenship, this study conducted a questionnaire survey to assess the current situation of cyberbullying among Chinese college students and examined the effect of students’ personal background, Internet use and social network habits, personality traits, emotions, and digital citizenship on cyberbullying from the perspective of individual students. The results showed that cyberbullying among college students is generally at a low level but still requires attention. At the personal background level, gender has a significant impact on college students’ cyberbullying and being cyberbullied, and the time to start using the Internet is significantly correlated to cyberbullying and being cyberbullied but has no significant impact on them. At the personal Internet use and social network habits level, the students’ average daily time online is not significantly correlated with cyberbullying and being cyberbullied; however, the proportion of online non-learning time is significantly positively correlated with cyberbullying, and the proportion of online learning/work time has a significant influence on students’ being cyberbullied. At the personality trait level, different Big Five personality traits have different correlations with and impacts on cyberbullying and being cyberbullied: openness is significantly positively correlated with cyberbullying and being cyberbullied; neuroticism and conscientiousness are significantly negatively correlated with cyberbullying and being cyberbullied; and agreeableness is significantly negatively correlated with cyberbullying. At the personal emotion level, life satisfaction is significantly negatively correlated with cyberbullying and being cyberbullied and has a significant impact on being cyberbullied; the personal stress and empathetic concern aspects of empathy are significantly positively correlated with cyberbullying and being cyberbullied among female students. At the personal digital citizenship level, students’ understanding of and compliance with Internet etiquette has a significant negative impact on cyberbullying, and digital communication and collaboration capabilities and Internet addiction have significantly positive impacts on cyberbullying and being cyberbullied; furthermore, their understanding of and compliance with digital laws and regulations is significantly negatively correlated with cyberbullying and being cyberbullied. Overall, college students’ digital citizenship level is significantly negatively correlated with cyberbullying but is not significantly correlated with being cyberbullied.

In this study, an attempt was made to explore the influencing factors of cyberbullying among college students, not only enriching the theory and practice of cyberbullying among students but also providing a new perspective for research in this field. Limited by several conditions, this paper only surveyed a small group of college students from modern cities in China. In a follow-up study, the sample size should be expanded as much as possible to provide more rational and reliable data support for drawing conclusions with a higher reference value. Furthermore, the effect of other levels such as the family, school, society, and the environment on cyberbullying should be taken into account so that comprehensive measures and governance processes can be developed to effectively curb cyberbullying among college students.

## Data Availability Statement

The original contributions presented in the study are included in the article/supplementary material, further inquiries can be directed to the corresponding author/s.

## Ethics Statement

Ethical review and approval was not required for the study on human participants in accordance with the local legislation and institutional requirements. Written informed consent for participation was not required for this study in accordance with the national legislation and the institutional requirements.

## Author Contributions

JZ: literature search, methodology, questionnaire survey, data analysis, and writing–review and editing. YZ: supervision, conceptualization, writing–original draft preparation, and review and editing. XH: literature search, questionnaire survey, and data analysis. DM and JG: literature search and questionnaire survey. ML: questionnaire survey and data analysis. JH: methodology and writing–revision and editing. All authors have read and agreed to the published version of the manuscript.

## Conflict of Interest

The authors declare that the research was conducted in the absence of any commercial or financial relationships that could be construed as a potential conflict of interest.

## Footnotes

1 View of world: The fundamental cognitive orientation of an individual or society encompassing the whole of the individual’s or society’s knowledge and point of view. View of life: The general and fundamental view of the purpose and meaning of life, the path of life and the way of life formed by people in practice. It determines the goal of people’s practical activities, the direction of life, and also the value orientation of people’s behavior choices and their attitude toward life. View of value: Cognitions, understandings, judgments, or choices made based on people’s certain thinking and senses. That is, a kind of thinking or orientation by which people recognize things and distinguish right from wrong.

## References

[B1] Al-Zahrani A. (2015). Toward digital citizenship: examining factors affecting participation and involvement in the Internet society among higher education students. *Int. Educ. Stud.* 8 203–217. 10.5539/ies.v8n12p203

[B2] Baldry A. C. Farrington D. P. Sorrentino A. (2015). “Am I at risk of cyberbullying”? A narrative review and conceptual framework for research on risk of cyberbullying and cybervictimization: the risk and needs assessment approach. *Aggress. Violent Behav.* 23 36–51. 10.1016/j.avb.2015.05.014

[B3] Barlińska J. Szuster A. Winiewski M. (2013). Cyberbullying among adolescent bystanders: role of the communication medium, form of violence, and empathy. *J. Community Appl. Soc. Psychol.* 23 37–51. 10.1002/casp.2137

[B4] Bayraktar F. Machackova H. Dedkova L. Cerna A. Ševčíková A. (2015). Cyberbullying: the discriminant factors among cyberbullies, cybervictims, and cyberbully-victims in a czech adolescent sample. *J. Interpers. Violence* 30 3192–32816. 10.1177/0886260514555006 25411234

[B5] Bevilacqua L. Shackleton N. Hale D. Allen E. Bond L. Christie D. (2017). The role of family and school-level factors in bullying and cyberbullying: a cross-sectional study. *BMC Pediatr.* 17:160. 10.1186/s12887-017-0907-8 28697725 PMC5505024

[B6] Beyazit U. Simsek S. Ayhan A. B. (2017). An examination of the predictive factors of cyberbullying in adolescents. *Soc. Behav. Pers.* 45 1511–1522. 10.2224/sbp.6267

[B7] Bottino S. M. B. Bottino C. M. C. Regina C. G. Correia A. V. L. Ribeiro W. S. (2015). Cyberbullying and adolescent mental health: systematic review. *Cad. Saúde Pública* 31 463–475. 10.1590/0102-311x00036114 25859714

[B8] Brack K. Caltabiano N. (2014). Cyberbullying and self-esteem in Australian adults. *Cyberpsychology* 8:7. 10.5817/CP2014-2-7

[B9] Brewer G. Kerslake J. (2015). Cyberbullying, self-esteem, empathy and loneliness. *Comput. Hum. Behav.* 48 255–260. 10.1016/j.chb.2015.01.073

[B10] Calvete E. Orue I. Estevez A. Villardon L. Padilla P. (2010). Cyberbullying in adolescents: modalities and aggressors’ profile. *Comput. Hum. Behav.* 26 1128–1135. 10.1016/j.chb.2010.03.017

[B11] Celik S. Atak H. Erguzen A. (2012). The effect of personality on cyberbullying among university students in Turkey. *Eurasian J. Educ. Res.* 49 129–150.

[B12] Chang F. C. Chiu C. H. Miao N. F. Chen P. H. Lee C. M. Chiang J. T. (2015). The relationship between parental mediation and Internet addiction among adolescents, and the association with cyberbullying and depression. *Compr. Psychiatry* 57 21–28. 10.1016/j.comppsych.2014.11.013 25487108

[B13] Chen J. K. Astor R. A. (2012). School variables as mediators of personal and family factors on school violence in taiwanese junior high schools. *Youth Soc.* 44 175–200. 10.1177/0044118x12448145

[B14] Chen L. Ho S. S. Lwin M. O. (2016). A meta-analysis of factors predicting cyberbullying perpetration and victimization: from the social cognitive and media effects approach. *New Media Soc.* 19 1194–1213. 10.1177/1461444816634037

[B15] China Internet Network Information Center (2020). *The 46th China Statistical Report on Internet Development.* Available online at: http://www.cac.gov.cn/2020-04/27/c_1589535470378587.htm (accessed February 3, 2021).

[B16] Davis M. H. (1980). A multidimensional approach to individual differences in empathy. *JSAS Cat. Sel. Doc. Psychol.* 10:80.

[B17] Del Rey R. Lazuras L. Casas J. A. Barkoukis V. Ortega-Ruiz R. Tsorbatzoudis H. (2016). Does empathy predict (cyber) bullying perpetration, and how do age, gender and nationality affect this relationship? *Learn. Individ. Differ.* 45 275–281. 10.1016/j.lindif.2015.11.021

[B18] Diener E. Emmons R. A. Larsen R. J. Griffin S. (1985). The satisfaction with life scale. *J. Pers. Assess.* 49 71–75. 16367493 10.1207/s15327752jpa4901_13

[B19] Elsaesser C. Russell B. Ohannessian M. C. Patton D. (2017). Parenting in a digital age: a review of parents’ role in preventing adolescent cyberbullying. *Aggress. Violent Behav.* 35 62–72. 10.1016/j.avb.2017.06.004

[B20] Festl R. Quandt T. (2013). Social relations and cyberbullying: the influence of individual and structural attributes on victimization and perpetration via the Internet. *Hum. Commun. Res.* 39 101–126. 10.1111/j.1468-2958.2012.01442.x

[B21] Floros G. D. Siomos K. E. Fisoun V. Dafouli E. Geroukalis D. (2013). Adolescent online cyberbullying in greece: the impact of parental online security practices, bonding, and online impulsiveness. *J. Sch. Health* 83 445–453. 10.1111/josh.12049 23586890

[B22] Gao T. (2018). *Investigation of the Practices of Australian OESCs to Maintain Internet Security for Elementary and Middle School Students.* Master’s thesis, Southwest University, Chongqing.

[B23] Gibb Z. G. Devereux P. G. (2014). Who does that anyway? Predictors and personality correlates of cyberbullying in college. *Comput. Hum. Behav.* 38 8–16. 10.1016/j.chb.2014.05.009

[B24] Giménez-Gualdo A. M. Hunter S. C. Durkin K. Arnaiz P. Maquilón J. J. (2015). The emotional impact of cyberbullying: differences in perceptions and experiences as a function of role. *Comput. Educ.* 82 228–235. 10.1016/j.compedu.2014.11.013

[B25] Gini G. Pozzoli T. (2009). Association between bullying and psychosomatic problems: a meta-analysis. *Pediatrics* 123 1059–1065. 10.1542/peds.2008-1215 19255040

[B26] Goodboy A. K. Martin M. M. (2015). The personality profile of a cyberbully: examining the dark triad. *Comput. Hum. Behav.* 49 1–4. 10.1016/j.chb.2015.02.052

[B27] Guarini A. Passini S. Melotti G. Brighi A. (2012). Risk and protective factors on perpetration of bullying and cyberbullying. Studia Educacyjne NR 23. *Studia Edukacyjne* 23 33–55.

[B28] Hao L. J. (2014). Theoretical analysis and ethical adjustment of college students’ online communication. *Audio Vis. Educ. Res.* 35 36–41. 10.13811/j.cnki.eer.2014.10.006

[B29] Hayton A. C. (2017). *Understanding Factors that Impact Cyberbullying Offending and Victimization.* Ph.D. dissertation, University of New Haven, New Haven, CT.

[B30] Hee O. C. (2014). Validity and reliability of the big five personality traits scale in Malaysia. *Int. J. Innov. Appl. Stud.* 5 309–315.

[B31] Hinduja S. Patchin J. W. (2008). Cyberbullying: an exploratory analysis of factors related to offending and victimization. *Deviant Behav.* 29 129–156. 10.1080/01639620701457816

[B32] Holland D. N. N. (2012). *The Internet Regression.* Available online at: http://www.psychomedia.it/pm/telecomm/telematic/holland2.htm (accessed January 15, 2012).

[B33] Hou M. H. Z. (2017). *Relationship between Internet Addiction and Cyberbullying Among Junior High School Students.* Ph.D. dissertation, Shaanxi Normal University Xi’an.

[B34] Howard P. J. Medina P. L. Howard J. M. (1996). “The big-five locator: a quick assessment tool for consultants and trainers,” in *The 1996 Annual*, ed. Pfeiffer J. W. (San Diego, CA: Pfeiffer & Company), 1–1310.

[B35] Hsu W. T. Wang C. H. (2010). *Investigation of the Correlation between Cyberbullying and Traditional Bullying among Middle School Students.* Master’s thesis, National Changhua Normal University Changhua.

[B36] Huang Y. Y. Chou C. (2010). An analysis of multiple factors of cyberbullying among junior high school students in Taiwan. *Comput. Hum. Behav.* 26 1581–1590. 10.1016/j.chb.2010.06.005

[B37] iiMedia Research (2018). *2018 Chinese College Students online Leisure Entertainment Behavior Monitoring Analysis Report.* Available online at: https://report.iimedia.cn/repo13-0/34426.html (accessed February 1, 2021).

[B38] Ivester M. (2011). *Lol…OMG!: What Every Student Needs to Know About Online Reputation Management, Digital Citizenship and Cyberbullying.* Reno, NV: Serra Knight Publishing.

[B39] Kiesler S. Zubrow D. Moses A. M. Geller V. (1985). Affect in computer-meditated communication: an experiment in synchronous terminal-to-terminal discussion. *Hum. Comput. Interact.* 1 77–104. 10.1207/s15327051hci0101_3

[B40] Kowalski R. M. Giumetti G. W. Schroeder A. N. Lattanner M. R. (2012a). Bullying in the digital age: a critical review and meta-analysis of cyberbullying research among youth. *Psychol. Bull.* 140 1073–1137. 10.1037/a0035618 24512111

[B41] Kowalski R. M. Limber S. P. Agatston P. W. (2012b). *Cyber Bullying: Bullying in the Digital Age*, 2nd Edn. Malden, MA: Wiley-Blackwell.

[B42] Li Q. (2007). New bottle but old wine: a research of cyberbullying in schools. *Comput. Hum. Behav.* 23 1777–1791. 10.1016/j.chb.2005.10.005

[B43] Liang F. (2019). How commonness and rational belief of cyber-bullying affect cyber-bullying behavior among middle school students: the mediated role of moral disengagement. *Psychol. Res.* 12 278–285.

[B44] Lin Y. (2017). *Study on the Prevention of Youth Cyberbullying from the Perspective of Digital Citizenship Education.* Master’s thesis, Zhejiang University of Technology, Hangzhou.

[B45] Liu Y. Xu W. (2019). The Psychological factors and treatment of cyberbullying. *Adv. Psychol.* 9 789–799. 10.12677/AP.2019.95097

[B46] Low S. Espelage D. (2013). Differentiating cyber bullying from non-physical bullying: commonalities across race, individual, and family predictors. *Psychol. Violence* 3 39–52. 10.1037/a0030308

[B47] Markward M. J. Cline S. Markward N. J. (2001). Group socialization, the Internet and school shootings. *Int. J. Adolesc. Youth* 10 135–146. 10.1080/02673843.2001.9747895

[B48] Monks C. P. Mahdavi J. Rix K. (2016). The emergence of cyberbullying in childhood: parent and teacher perspectives. *Psicol. Educ.* 22 39–48. 10.1016/j.pse.2016.02.002

[B49] Murwani E. (2019). Cyberbullying behavior patterns in adolescents in Jakarta. *J. Komunikasi Ikatan Sarjana Komunikasi Indonesia* 4 96–103. 10.25008/jkiski.v4i2.330

[B50] Nurlita F. Subagja A. R. R. Munawar H. Mulyani V. G. (2018). Analyzing factors affecting cyberbullying among students: its implication on social marketing. *Ind. Res. Workshop Natl. Semin.* 9 805–813. 10.35313/irwns.v9i0.1153

[B51] Ortega R. Elipe P. Mora-Merchán J. A. Calmaestra J. Vega E. (2009). The emotional impact on victims of traditional bullying and cyberbullying: a study of Spanish adolescents. *Z. Psychol.* 217 197–204. 10.1027/0044-3409.217.4.197

[B52] Ozden M. S. Icellioglu S. (2014). The perception of cyberbullying and cybervictimization by university students in terms of their personality factors. *Procedia Soc. Behav. Sci.* 116 4379–4383. 10.1016/j.sbspro.2014.01.951

[B53] Park S. Na E. Y. Kim E. M. (2014). The relationship between online activities, netiquette and cyberbullying. *Child. Youth Serv. Rev.* 42 74–81. 10.1016/j.childyouth.2014.04.002

[B54] Pawlikowski M. Altstötter-Gleich C. Brand M. (2013). Validation and psychometric properties of a short version of Young’s Internet Addiction Test. *Comput. Hum. Behav.* 29 1212–1223. 10.1016/j.chb.2012.10.014

[B55] Peluchette J. V. Karl K. Wood C. Williams J. (2015). Cyberbullying victimization: Do victims’ personality and risky social network behaviors contribute to the problem? *Comput. Hum. Behav.* 52 424–435. 10.1016/j.chb.2015.06.028

[B56] Peterson J. Densley J. (2017). Cyber violence: What do we know and where do we go from here? *Aggress. Violent Behav.* 34 193–200. 10.1016/j.avb.2017.01.012

[B57] Pillay C. L. (2012). *Behavioural and Psychosocial Factors Associated with Cyberbullying.* Master’s thesis, University of Zululand, Mhlathuze.

[B58] Renati R. Berrone C. Zanetti M. (2012). Morally disengaged and unempathic: Do cyberbullies fit these definitions? An exploratory study. *Cyberpsychol. Behav. Soc. Netw.* 15 391–398. 10.1089/cyber.2012.0046 22823490

[B59] Ribble M. (2015). *Digital Citizenship in Schools: Nine Elements all Students Should Know*, 3rd Edn. Washington, DC: International Society for Technology in Education.

[B60] Rosenberg M. (1965). *Society and The Adolescent Self-Image.* Princeton, NJ: Princeton University Press.

[B61] Safaria T. (2016). Prevalence and impact of cyberbullying in a sample of indonesian junior high school students. *Turk. Online J. Educ. Technol.* 15 82–90.

[B62] Schulte-Rüther M. Markowitsch H. J. Shah N. J. Fink G. R. Piefke M. (2008). Gender differences in brain networks supporting empathy. *Neuroimage* 42 393–403. 10.1016/j.neuroimage.2008.04.180 18514546

[B63] Semerci A. (2017). Investigating the effects of personality traits on cyberbullying. *Pegem Eğitim Öğretim Dergisi* 7 211–230. 10.14527/pegegog.2017.008

[B64] Silva A. D. Taveira M. C. Marques C. Gouveia V. V. (2015). Satisfaction with life scale among adolescents and young adults in portugal: extending evidence of construct validity. *Soc. Indic. Res.* 120 309–318. 10.1007/s11205-014-0587-9

[B65] Slonje R. Smith P. K. (2008). Cyberbullying: Another main type of bullying? *Scand. J. Psychol.* 49 147–154. 10.1111/j.1467-9450.2007.00611.x 18352984

[B66] Smith P. K. Mahdavi J. Carvalho M. Fisher S. Russell S. Tippett N. (2008). Cyberbullying: its nature and impact in secondary school pupils. *J. Child Psychol. Psychiatry* 49 376–385. 10.1111/j.1469-7610.2007.01846.x 18363945

[B67] Song Y. (2015). Cyberbullying and the responsibility of schools. *J. China Youth Coll. Polit. Sci.* 34 56–60. 10.16034/j.cnki.10-1318/c.2015.04.011

[B68] Sourander A. Klomek A. B. Ikonen M. Lindroos J. Luntamo T. Koskelainen M. (2010). Psychosocial risk factors associated with cyberbullying among adolescents. *Arch. Gen. Psychiatry* 67 720–728. 10.1001/archgenpsychiatry.2010.79 20603453

[B69] Souza S. B. Veiga Simão A. M. Ferreira A. I. Ferreira P. C. (2018). University students’ perceptions of campus climate, cyberbullying and cultural issues: implications for theory and practice. *Stud. High. Educ.* 43 2072–2087. 10.1080/03075079.2017.1307818

[B70] Sticca F. Ruggieri S. Alsaker F. Perren S. (2013). Longitudinal risk factors for cyberbullying in adolescence. *J. Community Appl. Soc. Psychol.* 23 52–67. 10.1002/casp.2136

[B71] Sun S. Deng S. (2016). Adolescents’ cyberbullying: causes, harms, and countermeasures. *Mod. Commun.* 38 144–148. 10.3969/j.issn.1007-8770.2016.02.026

[B72] Topcu C. Erdur-Baker O. (2010). The revised cyber bullying inventory (RCBI): validity and reliability studies. *Procedia Soc. Behav. Sci.* 5 660–664. 10.1016/j.sbspro.2010.07.161

[B73] Topcu C. Erdur-Baker O. (2012). Affective and cognitive empathy as mediators of gender differences in cyber and traditional bullying. *Sch. Psychol. Int.* 33 550–561. 10.1177/0143034312446882

[B74] Vranjes I. Baillien E. Vandebosch H. Erreygers S. De Witte H. (2017). The dark side of working online: towards a definition and an emotion reaction model of workplace cyberbullying. *Comput. Hum. Behav.* 69 324–334. 10.1016/j.chb.2016.12.055

[B75] Wang H. Zhou X. L. Lu C. Y. Wu J. Deng X. Q. Hong L. Y. (2012). Adolescent bullying involvement and psychosocial aspects of family and school life: a cross-sectional study from Guangdong Province in China. *PLoS One* 7:e38619. 10.1371/journal.pone.0038619 22815693 PMC3399853

[B76] Washington E. T. (2015). An overview of cyberbullying in higher education. *Adult Learn.* 26 21–27. 10.1177/1045159514558412

[B77] Weaver A. J. Lewis N. (2012). Mirrored morality: an exploration of moral choice in video games. *Cyberpsychol. Behav. Soc. Netw.* 15 610–614. 10.1089/cyber.2012.0235 23017118

[B78] Wong D. S. W. Chan H. C. Cheng C. H. K. (2014). Cyberbullying perpetration and victimization among adolescents in Hong Kong. *Child. Youth Serv. Rev.* 36 133–140. 10.1016/j.childyouth.2013.11.006

[B79] Yang H. Xu J. Zheng X. D. (2016). Digital citizenship in the information age. *China Educ. Technol.* 1 9–16. 10.3969/j.issn.1006-9860.2016.01.002

[B80] Ybarra M. L. Mitchell K. J. (2004). Youth engaging in online harassment: associations with caregiver-child relationships, Internet use, and personal characteristics. *J. Adolesc.* 27 319–336. 10.1016/j.adolescence.2004.03.007 15159091

[B81] You Y. (2013). *Questionnaire Revision and Influencing Factors Analysis of Cyberbullying Behavior.* Master’s thesis, Zhejiang Normal University, Hangzhou.

[B82] Zhang F. F. Dong Y. Wang K. (2010). Reliability and validity of the chinese version of the interpersonal reactivity Index-C. *Chin. J. Clin. Psychol.* 18 155–157. 10.3969/j.issn.1005-9202.2014.20.130

[B83] Zhao J. Z. Wang M. H. (2019). The relationship between self-presentation in social network and life satisfaction of undergraduates: intermediacy role of the self-concept clarity. *J. Baoding Univ.* 32 126–131. 10.13747/j.cnki.bdxyxb.2019.05.018

[B84] Zheng Y. X. Zhong J. P. Huang L. H. Yang H. (2020). Theoretical basis and training system of digital citizenship. *China Educ. Technol.* 5 69–79. 10.3969/j.issn.1006-9860.2020.05.017

[B85] Zhu H. Shi F. C. An L. Yin X. S. Fu M. H. Wang Y. D. (2016). Analysis on prevalence of cyberbullying in college students in China. *J. Jilin Univ. Med. Ed.* 42 605–611. 10.13481/j.1671-587x.20160337

